# Popperian ecology is a delusion

**DOI:** 10.1002/ece3.11106

**Published:** 2024-02-29

**Authors:** Jani Raerinne

**Affiliations:** ^1^ Faculty of Social Science University of Helsinki Helsinki Finland

**Keywords:** ecology, falsification, Popper, strong inference, testability

## Abstract

During the last 50 years, a group of ecologists has repeatedly used Popper's falsificationism in normative claims concerning how research in ecology should be conducted and/or how ecology should be corrected. Other ecologists seem to be dissatisfied with these criticisms. Nevertheless, they have not provided systematic analyses of how and why the Popperian criticisms of ecology fail. I have two aims in this article First, I show how so‐called Popperian ecologists have not only failed to use but have misused – if not abused – Popper in their criticisms of ecology. That is, the Popperian criticisms of ecology lack the justification the critics claim it has. Second, I claim that Popper's falsificationism is an unsuitable philosophy of science for ecology. In other words, ecology should not be criticized nor evaluated from the Popperian perspective in the first place.

## Abstract 2

Despite the fact that Popperphilia and Popperphobia have existed in ecology for more than 50 years, there has been no systematic and general critical review on Popper’s philosophy of science in ecology. The current ms. provides such a review. Such a review should have been published 30 years ago, since Popper’s falsificationism seems to be at best irrelevant to ecology.

## 1 INTRODUCTION

[T]he criterion of predictive power and falsifiability has been so widely embraced in ecology that critics accuse its supporters of ‘Popperphilia’… (Peters, 1991: 20–21)

Of all philosophies of science, Popper's (1965) falsificationism has been the most popular among ecologists. For instance, Peters (1976, 1978, 1991), Simberloff (1976, 1983), Connor and Simberloff (1979), Murray (1986, 1999, 2000, 2001), Loehle (1987), and Houlahan et al. (2017) claim to embrace many, if not all, of the central tenets of falsificationism. Of course, not all embrace falsificationism (e.g., Fagerström, 1987; Haila, 1982; Reiners & Lockwood, 2010; Turchin, 1999). Nevertheless, insofar as there is one philosophy of science that has been used as an exemplar to be defended or the main target to be criticized, it is clearly Popper's.

Popper's falsificationism has been popular, especially among ecologists criticizing ecology. Probably due to Peters' (1976, 1978) seminal papers, the discussion on falsificationism in ecology has centered on the problem of “tautology”, i.e., ecological hypotheses are pseudoscientific because central ecological concepts (e.g., stability, diversity, and competition) lack precision. I will provide a more comprehensive analysis than previous authors not only of ecology's status as a Popperian science but also of the suggestions by ecologists on how to improve ecology's status as a Popperian science. By ‘Popperian science or ecology’ I mean science/ecology that is falsificationist (i.e., deductive and non‐inductive).

In Section 2, I will describe the basic properties of a Popperian science. For so‐called Popperian ecologists, the science described in Section 2 is the point of reference against which the scientific status of ecology should be evaluated. In Section 3, I will compare ecology to a Popperian science. The comparison shows that ecology is the antithesis of a Popperian science. In contrast to what so‐called Popperian ecologists believe and suggest, however, the correct Popperian implication of the former is not that ecology is an immature science that should or could be improved, but a pseudoscience that should be eliminated instead. In Section 4, I will analyze the suggestions by ecologists on how to improve ecology's status as a Popperian science. As it turns out, their suggestions are typically anti‐ and non‐Popperian. Consequently, so‐called Popperian ecologists are not trying to turn ecology into a Popperian science. Instead, they are defending an ecology that is non‐ or anti‐Popperian. In Section 5, I will argue that Popperian ecology – were it ever to be practiced or realized – might not be as ideal as a science as so‐called Popperian ecologists tend to be believe.

While Popper has almost a monopoly in ecology insofar as ecologists are interested in philosophy of science, I am not suggesting that all or the great majority of ecologists are interested in Popper. There are ecologists who have little, if any, interests in philosophy. At the same time, many influential, prominent, and opinionated ecologists have been so‐called Popperians. These ecologists have published popular (text)books and articles that often criticize ecology (e.g., Murray, 1986, 1999, 2000, 2001; Peters, 1976, 1978, 1991; Simberloff, 1976, 1983 and Strong, 1980). Their publications have not only shaped the public image of ecology but have influenced later generations as well, given that Popper is even currently being discussed in the articles published in ecology journals. Compare the situation in ecology to evolutionary biology. Evolutionary biologists seem to have abandoned Popper already.

In what follows, I utilize ‘hypothesis’ for models, generalizations, laws, rules, and so on. The term is appropriate here, because it implies that the veracity of the items in question has not been established but needs to be tested.

## 2 POPPERIAN SCIENCE

Only the falsity of the theory can be inferred from empirical evidence, and this inference is a purely deductive one. (Popper, 1965: 55)

The critical attitude may be described as the conscious attempt to make our theories, or conjectures, suffer in our stead in the struggle for the survival of the fittest… We thus obtain the fittest theory within our reach by the elimination of those which are less fit… [B]y ‘fitness’ I do not merely mean ‘usefulness’ but truth… (Popper, 1965: 52)

I will briefly describe the basic ideas of falsificationism here (e.g., Popper, 1965). The summary is necessary, since ecologists do not apply Popper's ideas consistently and/or coherently. I will omit moderations of Popper's falsificationism by other authors (but see Lakatos, 1970; Loehle, 1987).

According to Popper, scientists should make bold conjectures. How we arrive at conjectures does not matter: they could be based on lucky guesses, myths, or observations. These conjectures, qua scientific hypotheses, should be stated as universally true statements or generalizations, such as that every instance of an X is also an instance of a Y in all time and space. Hypotheses stated as universally true generalizations (all Xs are Ys) are by themselves bold, since a single negative instance, such as a discovery of an X that is a non‐Y, establishes that they are false.

Bold hypotheses make risky or unforeseen predictions and/or have a high empirical content as well. More importantly, bold hypotheses make accurate predictions that are at least potentially testable and capable of being false. In other words, bold scientific hypotheses have a high degree of testability.

According to Popper, hypothesis testing aims to refute hypotheses. Thus we should be devising severe or crucial tests or experiments. If we come across even one negative instance to a hypothesis, we abandon the hypothesis, since universal hypotheses tolerate no exceptions. However, if no negative instances can be found, the hypothesis is not confirmed by positive instances; it is “corroborated.” Corroboration simply means that the hypothesis is tolerated – but not accepted – until it is refuted and replaced by a better hypothesis. Scientific progress, in turn, consists of eliminations: we come closer to the true(r) hypotheses by eliminating false ones and tentatively preserving corroborated hypotheses that have not (yet) been falsified.

Testability or falsifiability was not only suggested as the rational and critical scientific method, it was also presented as a criterion to demarcate science and pseudoscience. Pseudoscientific hypotheses tend to have high “explanatory” power: they can explain ex post facto all phenomena within their domain. Importantly, pseudosciences can explain cases that appear to falsify their hypotheses by introducing ad hoc hypotheses that save the original hypotheses from negative instances. Alternatively, pseudoscience makes such imprecise/vague predictions that they can be interpreted as being true no matter what the data shows. That is, only scientific hypotheses are testable and falsifiable, whereas pseudoscientific hypotheses are not.

Notice the difference between falsificationism and confirmationism. According to the latter, science proceeds by making predictions that are capable of confirming or verifying the hypotheses if the predictions turn out to be true. Popper was not a confirmationist, since he denied that induction had any rational justificatory role in science (hence also the difference between corroboration and confirmation). Only deductions can be trusted, since the knowledge deductions give is certain, whereas inductions are incapable of giving us certain knowledge. Why is this important or relevant? If hypotheses have a universal form (all Xs are Ys), we can never test the hypothesis for all of its potential instances. Thus, we can never be certain that the hypothesis is true despite how many positive instances it has, since it is possible that some of its instances in the past or future are negative. In contrast, even one negative instance of a hypothesis deductively falsifies it, since it is now established with certainty that the hypothesis was not true in all times and places. There is thus an asymmetry in the knowledge concerning hypotheses: we can be certain that a specific hypothesis is false when we encounter even one negative instance of it, whereas we can be never certain that the hypothesis is true regardless of how many positive instances it has. The true hypothesis is thus one that we never know is true: it is simply one that we have repeatedly failed to establish as false. In other words, hypotheses have only one known truth value: falsehood.

There is some confusion in the literature on the role of induction in the Popperian framework. Contrary to what some ecologists believe (e.g., Mentis, 1988), inductive methods and reasoning can be used to discover scientific hypotheses. Philosophers call this the context of discovery and differentiate it from the context of justification. For falsificationism, it does not matter how hypotheses are discovered. Falsificationism is only concerned with how hypotheses are justified (i.e., inductive methods/reasoning cannot be used to establish the truth or falsehood of hypotheses).

## 3 IS ECOLOGY A POPPERIAN SCIENCE?

I will provide here a comparison of ecology to a Popperian science. For so‐called Popperian ecologists, the falsificationist and deductive science described in the previous section is the benchmark against which the scientific status of ecology should be evaluated.

### 3.1 Ecology lacks universal hypotheses of the form “all Xs are Ys”

Consider the diversity‐stability rule as an example of a hypothesis that has universal form “in all communities, diversity (X) begets stability (Y).” Hardly any ecologist would present the rule in this way or form, however. The reason is that ecological hypotheses have exceptions. We know that there exist both monocultures that are very stable, such as saltmarshes, and very diverse communities that are unstable, such as intertidal ones. The hypothesis thus has many negative instances: Xs that are non‐Ys.

The problem is not that Popper demanded that scientific hypotheses have “unrestricted” universality, which shows that ecological hypotheses are false, given that ecological hypotheses typically apply to specific systems under specific conditions. For instance, Bergmann's rule does not apply to *all* organisms in *all* space and time. Rather, it states that the members of *endothermic* species are larger in body size in *colder regions* than the members of the same species in warmer regions. I believe that Popper would have agreed that the rule can be expressed so that it has the right properties of a scientific hypothesis, even though it has “restricted” universality: it applies to *all endothermic species*, ‘species’ refers to a class not to taxa, terms ‘endothermic,’ ‘body size,’ and ‘colder or warmer regions’ are valid scientific (theoretical) terms, and so on. The problem is that there are exceptions to the rule (i.e., endothermic taxa that do not follow the rule). That is, the rule is not true even within its more limited scope/domain. Many other ecological hypotheses (the island rule, Cope's rule, clutch size rule, etc.) can similarly be expressed as putative scientific hypotheses having “restricted” universality. Yet the problem is the same: they have exceptions. Moreover, since the above hypotheses are false even based on data we have on this planet and its systems, it would be pointless to speculate whether they would be true more universally (e.g., whether would they apply to similar extraterrestrial ecological systems under similar conditions).

Provided that we do not want to claim that nothing but already falsified hypotheses exist in ecology, hypotheses that have exceptions can be treated in at least two different ways. Both ways aim to save the hypotheses from their exceptions so that ecological hypotheses would be true.

First, one can try to maintain that hypotheses are qualified but universally true ones nevertheless, since they have the form “under typical or normal conditions, all Xs are Ys” (cf. Berryman, 2003). When conditions are not typical, negative instances are expected to happen. But these do not represent genuine exceptions, only apparent exceptions, since the hypotheses are not expected to hold under these conditions. At the same time, hypotheses are falsifiable and testable. If conditions are typical and a negative instance is encountered, the hypothesis is false, since in this case the negative instance represents a genuine exception.

The problem is that what is usual, normal, or typical cannot be stated in such a way that we have a closed set of conditions that would clearly state the conditions under which hypotheses are expected to hold. Competition theory provides an illuminating case. According to almost all the authors, the central prediction of competition theory is the competitive exclusion principle (or Gause's rule): n sympatric species with similar – let alone identical – niches cannot stably coexist for long periods of time on fewer than n number of resources. Over the last 100 years, we have discovered a complex and heterogeneous set of atypical conditions under which the competitive exclusion principle should not be expected to hold, however. What is even more worrisome is that the typical and non‐typical conditions themselves are stated imprecisely and loosely, such that *sufficient dissimilarities* between species could allow for coexistence, *intermediate* (a)biotic *disturbances* are capable of mediating the coexistence of competitor species, temporal or environmental *heterogeneity* could allow for coexistence, competing species cannot co‐exist *indefinitely*, and so on. We simply cannot state precisely and succinctly the conditions under which we should expect negative instances to falsify the predictions of competition theory (e.g., what exactly are disturbances and when exactly are they intermediate, and so on).

Consequently, and in practice, ecological hypotheses are qualified by nebulous ceteris paribus clauses, such as “all else being equal or if nothing interferes, all Xs are Ys.” The problem with ceteris paribus clauses is that they make hypotheses vacuously true. This not only drains hypotheses their empirical content but make them untestable as well. From the Popperian perspective, nebulously qualified hypotheses are thus not falsifiable nor scientific. Instead, they are pseudoscientific hypotheses.

The second strategy is to claim that ecological hypotheses are not universally but existentially true, such as “most or many of the Xs are Ys” (cf. Colyvan & Ginzburg, 2003). The problem is not the typical lament that hypotheses are not testable, because we are not given information on exactly what proportion of negative instances (5%, 15%, or 25%) would show them to be false. This is because the argument below applies even if we formulate our hypotheses as probabilistic laws, Pr(Y/X) = r, where r has some high but clearly stated value. The problem is that existentially true hypotheses cannot be falsified *at all within the Popperian framework*, because we cannot deductively falsify them. Consider a case in which our current tests/data reveal that only a few of the Xs are Ys, which is evidence against the hypothesis that most of the Xs are Ys. This would not suffice, however, since to make our knowledge concerning the falsity of the hypothesis certain, we would have to investigate all instances of Xs and Ys, including their instances in the past and future, since it might turn out that most of the Xs are Ys after all. Obviously, this cannot be done. Alternatively, we presume that uninvestigated Xs and Ys are similar to those already investigated. But this is not a valid strategy sensu Popper, since we are utilizing induction, not deduction. Again, the conclusion is that ecology is riddled with non‐falsifiable and non‐testable pseudoscientific hypotheses.

### 3.2 Lack of hypotheses making precise and risky predictions

Ecological hypotheses produce imprecise or qualitative (increases, decreases, oscillates, and so on) and conditional (Xs tend to be Ys; all Xs are Ys, when nothing interferes) predictions rather than stating exactly and precisely what must or will happen were the hypotheses true. The following provide examples: the number of species within a taxonomic group *typically increases* with *decreasing* latitudes; population growth rate *declines* with population numbers; there is a *tendency* for latitudinal ranges of species to become *smaller* with *decreasing* latitude; the abundant species *tend* to be *widely* distributed, while the rare species *tend* to have *restricted* ranges; and so on. A Popperian scientist would not be satisfied with such predictions, since they lack the testability and falsifiability of bold and accurate scientific hypotheses.

While it is generally accepted that ecology has a less than great record of making precise predictions (see Doak et al., 2008; Elliott‐Graves, 2019; Houlahan et al., 2017), there have been attempts to do so as well. A good example of a hypothesis providing precise and risky predictions was the 1.3 rule (Hutchinson, 1959), which has been falsified. What survived, however, was the hypothesis making safer and less precise qualitative predictions than the 1.3 rule, e.g., “*sufficient dissimilarities* could allow for the coexistence of competing species.” Another example is the species‐area rule, S = cA^z^, which also failed to provide exact and precise predictions in many cases. Again, what survived were imprecise and safer predictions, such as “isolated areas support *fewer* species than non‐isolated areas of similar size.”

When described in economic or financial terms, the difference is that risk aversion characterizes prediction making in ecology, whereas Popperian scientists are risk lovers.

### 3.3 Paucity of novel hypotheses in ecology

Ecologists have been conservative in postulating novel hypotheses (some exceptions, such as neutral theory, exist). When a new phenomenon or a novel target for explanations is encountered, such as a new data pattern, ecologists tend to re‐apply old hypotheses rather than coming up with novel ones. Over the last 50 or 60 years, a recurrent pattern has been that once a new phenomenon has been discovered, ecologists have tried to explain it in terms of competition or predation theories. Various phenomena have been “explained” in terms of competition or predation, such as the diversity and structure of communities, latitudinal gradients in body size, the latitudinal diversity gradient, the diversity‐stability hypothesis, the evolution of countless traits (e.g., body size), many phenomena about insular or isolated populations and communities, and the list goes on and on.

This conservativeness does not fit with the Popperian idea of progress. A Popperian science progresses by eliminating false hypotheses. But for this elimination to be possible, the scientific community must be able to produce novel alternative hypotheses that replace the falsified ones. Ecology seems to lack the Popperian potential to progress toward the truth. Instead, ecology has stagnated, since it seems to be recycling old hypotheses. Publication data collected from journals also support the idea that a few theories/hypotheses dominate research in ecology (see Raerinne, 2023).

Note that I do not claim that ecology lacks “fads” (cf. Paine, 2002). Various specific hypotheses, methods, techniques, and so on have been popular in ecology during different times, after which they have vanished. Consider Clementsian succession theory, null models/hypotheses during the 1980s, and the currently popular facilitation theory as examples of fads. Fads are not *novel alternative* hypotheses in the above Popperian sense. Fads are typically specific “expressions” of old dominant hypotheses (succession theory and competition), specific “expressions” of old but less dominant hypotheses (facilitation theory and mutualism), or short‐lived critical reactions to dominant hypotheses that fail to change the status quo (null models and competition theory).

### 3.4 Refusals to refute

Ecologists tend to explain away exceptions to their hypotheses. The most famous case concerns the “paradox of the plankton” by Hutchinson (1961).

Hutchinson (1961) discovered that there exist many cases in which sympatric phytoplankton species with similar or even identical niches coexisted for long periods of time even though the number of their resources was clearly less than the number of coexisting species. The case was clearly the opposite to what the competitive exclusion principle predicts.

Rather than trying to falsify the competitive exclusion principle, let alone competition theory, Hutchison explained the negative instance away using temporal environmental heterogeneity and non‐equilibrium communities. In short, Hutchison claimed that the negative instance is not a genuine exception, but an apparent exception, since the coexistence is not stable.

Hutchinson (1961) has become a classical reference in ecology. However, the case must be evaluated from the Popperian perspective. Without a doubt, any consistent Popperian should condemn Hutchinson's strategy as a perversion of a Popperian science. Note that the issue is not that Popper would have claimed that *all* negative instances falsify hypotheses. We know that spurious negative instances exist. Spurious negative instances could arise, for instance, if the test were deficient or if we have made errors in data collection or analysis. The situation here is different. First, Hutchinson had no prior empirical/theoretical evidence that his explanation for the negative instance was true. Nor did he claim that the data/results/tests were deficient. Rather, Hutchinson presented a genuine ad hoc hypothesis, which had one purpose: to save another hypothesis from its falsification. Second, this practice of explaining away exceptions is common in ecology (see Raerinne, 2017 and references therein).

Note that I did not claim that ecologists disregard or shun negative instances. Rather, ecologists acknowledge the exceptions and actively try to account for them. Nevertheless, from the Popperian perspective, this shows that ecologists do not (mainly) seek falsifications and refutations, but (mainly) seek confirmations.

A Popperian science is innovative and bold in its character, riddled with testable and falsifiable hypotheses making universal, risky, and precise predictions. A Popperian science deductively progresses toward the truth by eliminating false hypotheses and replacing these with novel ones.

What kind of a science is ecology? Ecology is a timid and conservative science: it recycles and reapplies existing hypotheses, which make existential/qualified, safe, and imprecise predictions. Ecology progresses toward the truth inductively by trying to verify the truth of a few dominant hypotheses and saving the hypotheses from falsification when possible.

Were so‐called Popperian ecologists consistent and coherent Popperians, the conclusion seems to be that ecology is an antithesis of a Popperian science: a pseudoscience, which does not seem to have many, if any, of the characteristics that Popper postulated for the sciences. The correct Popperian implication is not the correction but the *elimination* and replacement of ecology with a completely different science, in contrast to what so‐called Popperian ecologists seem to believe and suggest.

Even if the above shows that a perfect Popperian ecology might be an unattainable goal, the positive side for so‐called Popperian ecologists is that the discussion has revealed various shortcomings that could be corrected to make ecology at least *more* Popperian science.

What has been claimed in this section was not presented as a criticism of ecology per se. Instead, discussion was comparative: ecology seems to lack the properties of a Popperian science.

## 4 SO‐CALLED POPPERIAN ECOLOGISTS ARE NOT REAL POPPERIAN SCIENTISTS

I will discuss here the main suggestions by ecologists for how to improve ecology's status as a falsificationist science and consequently to make ecology at least more Popperian as a science. I will omit Peters' (1976, 1978) early discussion on the tautology problem and his suggestions for how to improve conceptual precision in ecology (and evolution). Previous authors have already shown why Peters' version of the tautology problem was erroneous (e.g., Caplan, 1977; Shrader‐Frechette, 1990). Moreover, there is enough discussion in the literature on whether ecological concepts, such as competition or stability, are imprecise, conflated, or “tautologies” (see Cole, 1960; Grimm & Wissel, 1997; McCoy & Shrader‐Frechette, 1992; Shrader‐Frechette, 1990, and references therein). Also, I will omit here ecologists who discuss ideas they believe Popper advocated (e.g., Houlahan et al., 2017 and many others), but do not systematically and repeatedly utilize Popper to criticize ecology, as the authors discussed below have done. It is beyond the topic of this article to provide discussion on all the various and sometimes conflicting ideas ecologists have interpreted to be Popperian.

So‐called Popperian ecologists sometimes equate falsificationism with instrumentalism. According to instrumentalism, hypotheses (or theories) are interpreted to be tools to make practical predictions rather than giving us theoretical or causal explanations. According to Peters (1991: chapter 5), instrumentalism helps to make ecology more testable, since causal terminology is vacuous, which makes causal and mechanistic hypotheses untestable/non‐falsifiable. Consequently, we should focus on correlations and regressions (e.g., allometries and scaling laws), which are (more) testable and falsifiable. I shall here not dwell on Peters' erroneous belief that causal terminology is vacuous or untestable; see Woodward (2003) for a testable and non‐tautologous account of causation.

As a realist, Popper rejected instrumentalism. Peters is aware of this as well, but since Peters fails to respect the reasons why Popper (1965: 111–114) deemed instrumentalism incompatible with falsificationism, I will re‐state these here. If hypotheses are treated as tools, they cannot be falsified on the basis of their *instrumentalistic* value. Typically tools are not either applicable or inapplicable, but *more or less* applicable. Moreover, false hypotheses often function as successful predictive tools. For instrumentalism, false but useful hypotheses are thus acceptable, whereas falsificationism tries to find true hypotheses by eliminating all the false ones regardless of their practical value (see also the quotation from Popper in Section 2). Instrumentalistic ecology would thus not be more Popperian but an *anti*‐Popperian science.

It is true that Popper's criterion of testability does emphasize predictive power, which is probably one reason why some ecologists, including Peters (1991), have equated falsificationism with what could be called predictive and correlative ecology. Nevertheless, Popper did not deem correlations more scientific or testable than causal or explanatory hypotheses, as Peters (1991) suggests. In fact, it could be argued that causal hypotheses are more testable within the Popperian framework than correlations in that the former give us more exact and risky knowledge about the conditions under which the hypotheses could be falsified. If X is a cause of Y, then the two should not be effects of a common cause Z. Moreover, systematic variations in the value of the cause variable X should produce systematic changes in the value of the effect variable Y, and not vice versa. In the cases of correlations, however, it does not matter whether X is a cause of Y, Y is a cause of X, or whether the two are common effects of Z. None of the three alternatives falsify the hypothesis postulating correlation, whereas the causal hypothesis is falsified by two cases out of the three. The upshot is that causal hypotheses are more scientific, testable, and falsifiable than correlations/regressions, in contrast to what Peters (1991) believes. Peters' (1991) call for predictive and non‐causal ecology would thus make ecology *less* Popperian as a science.

Some, but not all, null modelers explicitly justify their methodology by referring to Popper (e.g., Connor & Simberloff, 1979; Simberloff, 1976, 1983). Since Sloep (1986, 1991) has already shown why falsificationism is irrelevant in this context, I will make my own points succinctly. Null modelers claim that hypotheses should not be tested alone, but we should also investigate how alternative hypotheses – so‐called null hypotheses – could fit the same data. While there have been debates over what null hypotheses are, the basic idea is simple. A hypothesis and its null hypothesis should be exclusive alternatives that are compared against a common set of test data. The basic claim of null modelers is that if a null hypothesis can be accepted (e.g., the null hypothesis fits statistically well with common test data), then the *confirmation* of the null hypothesis implies the rejection (or the irrelevance) of the hypothesis to which the null hypothesis was presented as an exclusive alternative. This shows that the null hypothesis method is confirmationist, not falsificationist. A Popperian scientist would have tried to *falsify both hypotheses*.

**FIGURE 1:**
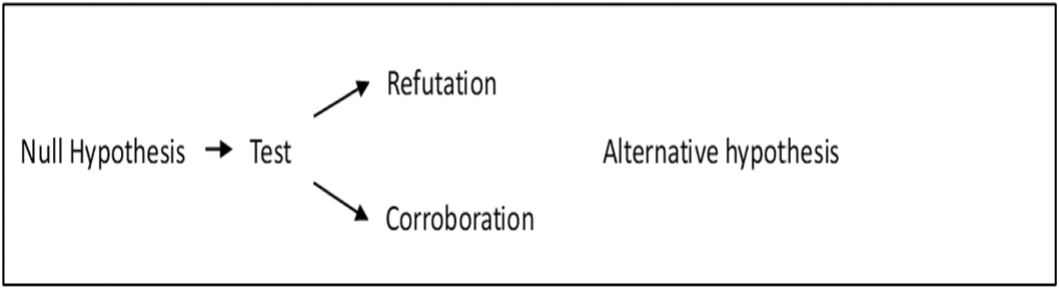
Falsificationism and deductive null hypothesis testing. Corroboration of a null hypothesis implies nothing about the truth value of a hypothesis that is its exclusive alternative, since corroborated hypotheses have no truth values. The only way to establish the truth value of the alternative hypothesis is to test it independently (i.e., to falsify it).

Null modelers referred to above have been excellent illusionists in using terminology that give the program a Popperian appearance. A null modeler could object to my claim that they do not hold that null hypotheses are accepted or confirmed. Instead, they claim that if the null hypothesis fails to be rejected or falsified based on a certain statistical test, such as p value, then the falsehood (or irrelevance) of the other hypothesis can be inferred (cf. Strong, 1980: 280 “failure to disprove the null hypothesis does not prove it”). Consequently, they are not confirmationists, but falsificationists, since they are making (statistical) claims about the failure to reject a hypothesis rather than its acceptance or truth. Let me reiterate, however, that in the Popperian context “fails to be rejected or falsified” can only mean that the hypothesis is a pseudoscientific hypothesis (i.e., it cannot be properly tested), a hypothesis that has not yet been tested (i.e., it can be tested), or a corroborated and already tested hypothesis (many ecologists believe erroneously that corroboration means confirmation; see Section 2 for the difference). In all cases, however, the hypothesis has no known truth value, since within the Popperian framework hypotheses can have only one known truth value: falsehood. A hypothesis that has no known truth value imply nothing about the truth value of another hypothesis, even if the two are exclusive alternatives (cf. Figure 1). Thus, the *logic* of null modelers *itself* requires that they are not Popperians and thus that “non‐rejected” or “non‐falsified” means in practice accepted or confirmed.

**FIGURE 2:**
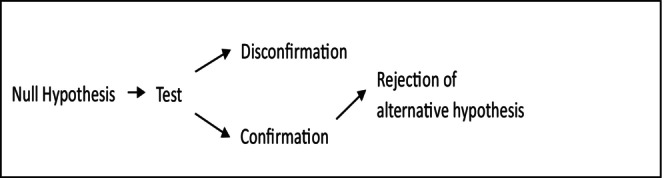
Null hypothesis testing under inductivism, including ‘strong inference.’ The truth value of a (well) confirmed hypothesis implies that the hypothesis that is its exclusive alternative must be false. Note that for the implication to hold, the hypotheses must be contradictory. If hypotheses are only so‐called contraries, the above implication does not hold, since both could be false.

The method of null modelers is in fact a variant of what Platt (1964) called ‘strong inference.’ Despite the erroneous belief of many authors that strong inference is a falsificationist method (e.g., Loehle, 1987; Mentis, 1988; Simberloff, 1983: 627), what Platt had in mind was the scientific method that Popper rejected and attacked. Platt is defending a Baconian *inductive* and *confirmationist* method utilizing crucial experiments to select the true hypothesis among competing ones. The truth of one hypothesis – the one which the experiment confirmed – implies the falsehood of its competitors (see Figure 2). What the null modelers are thus defending is not a Popperian, but an anti‐Popperian hypothesis testing in ecology.

What has just been claimed should not be taken to suggest that null models have no merits. I only argued that the method should not be defended on Popperian grounds, as some of the critics of traditional hypothesis testing in ecology have done. A Popperian version of null model hypothesis testing would simply lack the merits the proponents of the method claim it has. Since Zhang (2020) has already discussed other virtues and vices of null models that are not related to falsificationism, I shall not repeat these here. There is, however, one topic that has gone undiscussed in this context, namely, what is the epistemic or evidential relevance of simulated data null models produce (see Lehtinen & Raerinne, 2023 for a general discussion on simulated data in empirical science).

Even perhaps the most consistent of Popperians, Murray (1986, 1999, 2000, 2001), admits that induction or confirmation plays a justificatory role in ecology (e.g., Murray, 1999: 594). As the discussion has already shown, Murray is not alone here. This is no minor relapse, however. With induction the basic justification for falsificationism disappears. What we have, instead, is again some variant of Platt's (1964) strong inference (which seems to be case in Murray, 1986). What we do not have, again, is falsificationism.

My claim is not that so‐called Popperian ecologists fail to be *naïve* or *strict* Popperians. My claim is that they are not Popperians at all. First, so‐called Popperian ecologists fail to include the proprietary Popperian property of science – that hypotheses can only be deductively falsified, never inductively confirmed – into their methods. Second, their suggestions for how to improve ecology's status as a falsificationist science do make ecology not more, but less Popperian, or even anti‐Popperian as a science. Third, so‐called Popperian ecologists seem to be demanding some of the right, but very general properties from ecology (terms should be defined accurately, the predictions of hypotheses should be stated clearly and tested rigorously, crucial experiments should be used to eliminate competing hypotheses, and so on) but for the wrong reasons. First, what so‐called Popperian ecologists demand are not proprietary Popperian properties of science, since inductivists have demanded these from science centuries before Popper. Their demands have thus nothing to do with Popper's falsificationism. Second, some of the Popperian suggestions in this context backfire as well. A Popperian variant of the method of null models would probably make ecology less testable and less rigorous in hypothesis testing. This variant lacks the merits the proponents of the method claim null models have, in contrast to the non‐Popperian variant of the method (see Figures 1 and 2). At the same time, so‐called Popperian ecologists seem to be demanding some questionable, idiosyncratic, or debatable properties from ecology as well (e.g., that ecologists should focus on correlations only). Again, however, these demands have nothing to do with Popper's falsificationism. Finally, it seems that many so‐called Popperian ecologists are not worried about the testability of hypotheses in general, which would be a Popperian concern. Instead, they are worried about the status of certain dominant and specific hypotheses only – especially those that are related to competition theory (cf. Connor & Simberloff, 1979; Peters, 1976, 1978, 1991; Simberloff, 1976, 1983). However, this seems to be a Kuhnian theme and concern about paradigms (Kuhn, 1962). In short, so‐called Popperian ecologists have not only failed to use Popper but have misused – if not abused – Popper in their criticisms of ecology.

## 5 WOULD POPPERIAN ECOLOGY BE A GOOD SCIENCE?

Since so‐called Popperian ecologists criticize ecology from the Popperian perspective, this implies that they believe that Popperian ecology would be a good or ideal science and/or that falsificationism helps to make ecology a better science. I will, however, suggest that Popperian ecology could be epistemically and ethically a suspicious science. In other words, falsificationism would make ecology worser as a science. This shows that so‐called Popperian suggestions for how to improve ecology's status as a science do not just lack justification, they lack motivation as well.

A Popperian ecologist should focus on investigating the conditions under which hypotheses fail rather than trying to discover conditions in which hypotheses might succeed and work. A Popperian ecologist should also disregard the evidential support of hypotheses. This would create acute issues, especially in policy‐ or decision‐making contexts. In these contexts, one of the main issues that an ecologist faces is to select the right hypothesis (or a few of them) among the competing ones to be recommended that has the best evidential support to be applicable to the cases at hand. (There are other practical, social, legal, economic, and ethical issues that need to be considered in these contexts. I will focus on certain epistemic and evidential issues only).

What hypothesis would a Popperian ecologist recommend to policy‐ or decisionmakers? Obviously, the answer is the one that has been most rigorously tested (i.e., the most corroborated hypothesis). However, this would not amount to selecting the right hypothesis. This is since the most relevant information in this context (which hypothesis has the best evidential support to be applicable to the cases) is deemed as epistemically and evidentially irrelevant by a Popperian ecologist, whereas the epistemically and evidentially more irrelevant or supplementary information (the falsity of a hypothesis to other, dissimilar cases; see below) is deemed as the only relevant information by a Popperian ecologist.

The problem is the same we encountered in Section 3, namely, that ecological hypotheses have negative instances. The hypothesis having the best evidential support to be applicable to the cases is probably one that has been shown to be false with regard to other cases that are dissimilar to the cases at hand (e.g., while the diversity‐stability rule might apply to many communities, including the cases at hand, intertidal communities and saltmarshes are exceptions to the rule). Consequently, from the Popperian perspective, the right hypothesis has already been refuted if it is expressed as a “all Xs are Ys” hypothesis. Or the right hypothesis cannot be recommended, if it is expressed as a qualified or existential hypothesis (“ceteris paribus, all Xs are Ys” or “most of the Xs are Ys”), which tolerate exceptions, since these are pseudoscientific hypotheses within the Popperian framework.

An obvious reply to above claims is that ecologists are common sense Popperians. In theory, Popper might have claimed that we can never accept nor confirm well‐corroborated hypotheses, including those that happen to be true. We simply have no other choices but to tolerate true hypotheses ad infinitum, since we cannot never establish that they are true or false. In practice, however, everybody agrees that well‐corroborated hypotheses have some confirmation to be true or applicable. If the right hypothesis is thus well‐corroborated and has not been shown to be false with regard to cases that are similar to the cases at hand, it can and should be recommended, despite the fact that all ecological hypotheses have some other negative instances, including the right hypothesis. The difference to the case discussed above is that the most relevant information (which hypothesis has the best evidential support to be applicable to the cases) is no longer complete irrelevant nor is the more irrelevant or supplementary information (the falsity of a hypothesis to other, dissimilar cases) as the only relevant information in the case of a common sense Popperian. I am skeptical that this would help much, however. A scientist without a cognitive negativity bias is no Popperian – common sense or not. Negative instances trump positive ones. Falsification trumps confirmation. The case discussed above would now be analogous to an absent‐minded dealer of used cars who is unable to close deals, since (s)he remembers and mentions only or mainly cars' flaws, defects, and so on.

Ecologists' interests in Popper's falsificationism would be understandable were it the case that falsificationism was the received view in philosophy and/or suitable to ecology. But falsificationism is neither. For instance, the conclusion one could draw from the fact that ecology lacks the properties of a Popperian science (see Section 3) is not necessarily that ecology is a pseudoscience, but that falsificationism fails to account for the scientific status of ecology. In other words, falsificationism seems to be unsuitable philosophy of science for ecologists in the first place. Luckily, there are more suitable philosophies of science for ecologists (cf. Cooper, 2003; Elliot‐Graves, 2023; Justus, 2021 and Wimsatt, 2007). Finally, while the infamous physics envy could partly explain the popularity of Popper in ecology (i.e., falsificationism helps to transform ecology into a mature science similar to physics), it is debatable whether (even) physics have the properties of a Popperian science.

## 6 CONCLUSIONS

Ecology's harshest critics have been ecologists themselves. Popper's falsificationism has been popular especially among ecologists criticizing ecology. So‐called Popperian ecologists have failed to base and motivate their criticism of ecology on Popper's falsificationism, however. First, Popper's falsificationism does not seem to imply that ecology should or could be improved, as so‐called Popperian ecologists believe, but that ecology should be eliminated instead. Second, the suggestions of so‐called Popperian ecologists for how to improve ecology's status as a Popperian science do not make ecology more, but less and even anti‐Popperian as a science. Third, Popperian ecology – were it ever to be realized or practiced – could be epistemically and ethically a suspicious science. Its omissions (incapacity to deliver positive policy recommendations, instruments, or recipes) and biased knowledge base (on conditions under which hypotheses fail) would probably cause harm to ecology, society, and the environment. Moreover, it seems that falsificationism is neither able nor needed to fix any of the existing problems in ecology (e.g., testability). Fourth, falsificationism seems to be unsuitable philosophy of science for ecologists in the first place. It fails to account for the scientific status of ecology, suggesting that ecology is a pseudoscience instead.

I have not suggested that ecology should not be criticized. I have only argued that ecology should not be criticized nor evaluated from the Popperian perspective.

Popperphilia and Popperphobia have existed in ecology for more than 50 years. Perhaps it is time to move on to other philia and phobia, since, first, Popperian ecology seems to be a delusion and, second, the fishing for (more) Popperian ecology has caught mainly red herrings.

## AUTHOR CONTRIBUTIONS

**Jani Raerinne:** Conceptualization (lead); data curation (lead); formal analysis (lead); funding acquisition (lead); investigation (lead); methodology (lead); project administration (lead); resources (lead); software (lead); supervision (lead); validation (lead); visualization (lead); writing – original draft (lead); writing – review and editing (lead).

## FUNDING INFORMATION

Emil Aaltonen Foundation (project no. 230166 N1V).

## CONFLICT OF INTEREST STATEMENT

The author has no conflicts of interest to report.

## Data Availability

Not applicable.
